# Functional Comparison between Healthy and Multiple Myeloma Adipose Stromal Cells

**DOI:** 10.1155/2020/4173578

**Published:** 2020-03-05

**Authors:** Nicolas Espagnolle, Benjamin Hebraud, Jean-Gérard Descamps, Mélanie Gadelorge, Marie-Véronique Joubert, Laura Do Souto Ferreira, Murielle Roussel, Anne Huynh, Luc Sensébé, Louis Casteilla, Michel Attal, Hervé Avet-Loiseau, Frederic Deschaseaux, Philippe Bourin, Jill Corre

**Affiliations:** ^1^STROMALab, Université de Toulouse, CNRS ERL5311, EFS, INP-ENVT, Inserm U1031, UPS, Toulouse, France; ^2^EFS Occitanie, 31042 Toulouse, France; ^3^Hematology Department, Institut Universitaire du Cancer, Toulouse, France; ^4^Laboratory for Genomics in Myeloma, Institut Universitaire du Cancer, Toulouse, France; ^5^Centre de Recherche en Cancérologie de Toulouse INSERM U1037, Toulouse, France; ^6^Cell-Easy, Centre Pierre Potier, 31106 Toulouse Cedex 1, France

## Abstract

Multiple myeloma (MM) is an incurable B cell neoplasia characterized by the accumulation of tumor plasma cells within the bone marrow (BM). As a consequence, bone osteolytic lesions develop in 80% of patients and remain even after complete disease remission. We and others had demonstrated that BM-derived mesenchymal stromal cells (MSCs) are abnormal in MM and thus cannot be used for autologous treatment to repair bone damage. Adipose stromal cells (ASCs) represent an interesting alternative to MSCs for cellular therapy. Thus, in this study, we wondered whether they could be a good candidate in repairing MM bone lesions. For the first time, we present a transcriptomic, phenotypic, and functional comparison of ASCs from MM patients and healthy donors (HDs) relying on their autologous MSC counterparts. In contrast to MM MSCs, MM ASCs did not exhibit major abnormalities. However, the changes observed in MM ASCs and the supportive property of ASCs on MM cells question their putative and safety uses at an autologous or allogenic level.

## 1. Background

Multiple myeloma (MM) is a B cell neoplasia that accounts for approximately 1% of all cancers and 10% of hematologic neoplasias, with median age at diagnosis of about 70 years [1, 2]. MM is characterized by the accumulation of tumour plasma cells/myeloma cells (MM cells) within the bone marrow (BM) and the production of monoclonal protein in serum and/or urine. Osteolytic bone disease develops in more than 80% of MM patients [3] and often leads to severe bone pain and pathologic fractures [4, 5]. These irreversible symptoms have a huge impact on morbi-mortality in MM [6], resulting from excessive osteoclastic bone resorption and inhibited osteoblastic bone formation.

In recent decades, the key role of BM-derived mesenchymal stromal cells (MSCs) in survival and drug resistance of MM cells has been well documented [7–10]. More than 10 years ago, we hypothesized that autologous MSCs could be used in autologous stem cell transplantation for MM treatment because of their ability to differentiate to osteoblasts and support hematopoiesis [11]. Unfortunately, we and others have demonstrated that MSCs are abnormal in MM [12–14]; in particular, they produce not only excess MM growth factors such as interleukin-6 (IL-6) [15] but also growth and differentiation factor 15 (GDF15), which is also responsible for chemoprotection [8]. Importantly, their ability to differentiate in osteoblasts is severely impaired, even without any contact with MM cells [16], one explanation being their abnormally high secretion of the Wnt inhibitor Dickkopf 1 (DKK1).

Distant from the pathologic medullar microenvironment, adipose tissue is an easily accessible and enriched source of adipose stromal cells (ASCs), representing an interesting alternative to MSCs for cellular therapy [17]. ASCs have comparable properties to MSCs in the ability to differentiate *in vitro* to mesoderm lineages, especially osteoblastic pathway, and to support hematopoiesis [18–20].

To test the potential use of ASCs as a cell therapy product for counteracting the irreversible bone lesions in MM, we compared the behaviour of ASCs and MSCs in a physiological and pathological context. This work presents a transcriptomic, phenotypic, and functional comparison of ASCs and MSCs from the same MM patients or healthy donors (HDs) to determine whether ASCs are suitable for treating bone disease in MM.

## 2. Materials and Methods

### 2.1. Participants

ASCs and MSCs were from 12 MM patients and 12 allogenic BM donors (HDs). The Comité de protection des personnes (CPP sud-ouest et Outremer I) approved the study, and written informed consent was obtained from all patients included. All MM patients have been diagnosed in the Hematology Department of Institut Universitaire du Cancer de Toulouse in France.

### 2.2. Cells

BM was aspirated by sternal puncture for MM patients and from the posterior iliac spine for HDs. BM cells were seeded at 5.10^4^ cells/cm^2^ in complete medium (Minimum Essential Medium-*α*, Life Technologies, Carlsbad, CA, USA) supplemented with 10% fetal calf serum (Lonza, Levallois-Perret, France) and 10 *μ*g/ml ciprofloxacin (Bayer, Puteaux, France). The medium was renewed twice a week until cells were confluent (P0) and reseeded until confluence (P1) [21, 22]. ASCs were isolated from the stromal vascular fraction of subcutaneous adipose tissue (periumbilical area for MM patients and iliac spine for HDs) after enzymatic digestion. The same culture conditions as for MSCs were used.

Hematopoietic CD34+ cells were sorted by using CD34 microbeads on granulocyte colony stimulating factor-mobilized blood from MM patients (Miltenyi Biotec, Bergisch Gladbach, Germany).

The MOLP6 stroma-dependent MM cell line was a generous gift from Dr. Harashima (Fujisaki Cell Center, Hayashibara Biochemical Laboratories, Japan). Cells were grown on MSC stroma in RPMI medium-1640 (Life Technologies) with 10% fetal calf serum and 10 *μ*g/ml ciprofloxacin [23].

### 2.3. Transcriptomic Analysis

ASC mRNA was extracted by using the RNeasy Kit (Qiagen, Hilden, Germany). Biotinylated cRNA synthesis, hybridization to human U133 plus 2.0 GeneChip microarrays (Affymetrix, Santa Clara, CA, USA), and analysis were performed as described [24]. Data were deposited in the GEO dataset in Medline: accession number *GSE133346*.

### 2.4. Differentiation Assays

#### 2.4.1. Toward Osteoblastic Lineage

ASCs were seeded at 5000 cells/cm^2^ and incubated with complete medium, complemented with 0.1 mM dexamethasone and 50 *μ*M ascorbic acid (Sigma, Lyon, France) for 21 days. For mineralization assay, 3 mM inorganic phosphate was added during the first 14 days and *β*-glycerophosphate 10 mM during the final 7 days. At day 21, alkaline phosphatase activity was quantified by fluorescent measurement of its fluorescent substrate Attophos (Promega, Charbonnières, France). Mineralization assay was performed with alizarin red staining, as described [25].

#### 2.4.2. Toward Adipocytic Lineage

ASCs were seeded at 20000 cells/cm^2^ and incubated with complete medium supplemented with 1 *μ*M dexamethasone, 0.45 mM isobutyl methylxanthine (IBMX), and 60 *μ*M indomethacin. At day 21, triglycerides were measured by using the TG PAP 150 kit, following the supplier's recommendations (bioMerieux).

#### 2.4.3. Toward Chondrocytic Lineage

A total of 250000 ASCs were seeded as a pellet in a nonadherent tube in complete medium with 0.1 *μ*M dexamethasone, 1 1% ITS+, 1 mM pyruvate sodium, 0.17 mM ascorbic-2-phospate acid, and 0.35 mM L-proline. At day 18, pellets were digested for 4 h at 56°C with a solution of 1 mg/ml proteinase K, plus 10 *μ*g/ml A pepstatin, 1 mM iodoacetamide, and 1 mM EDTA. Glycosaminoglycan dosage was measured by absorbance (525 nm) immediately after mixing the digestates with 16 *μ*g/ml dimethylmethylene blue solution, plus 3 mg/ml glycine and 2.4 mg/ml NaCl. A range of sulfate chondroitin was measured in parallel.

### 2.5. Support of Hematopoiesis Assessment

An amount of 4.10^4^ hematopoietic CD34+ cells was seeded in coculture with 2.10^4^ ASC stroma in 2 ml MyeloCult medium containing 1 *μ*M hydrocortisone (Stem Cell Technologies, Grenoble, France) in 12-well plates. At days 7, 14, 21, and 28, half of the medium was counted for nonadherent cells and assayed for hematopoietic progenitors in methylcellulose (Miltenyi Biotec). The total number of colonies from nonadherent cells in the coculture was evaluated.

### 2.6. Assessment of Support of Myeloma Cell Growth

HD/MM ASCs or MSCs were seeded in 12-well plates at 11250 cells/cm^2^. After 24 h of culture, 2.10^4^ MOLP-6 cells were added to each well with 1 ml complete RPMI medium. The number of MOLP-6 cells was evaluated on day 7.

### 2.7. Immunophenotype

HD/MM ASCs or MSCs were stained with fluorochrome-conjugated anti-CD73/CD90/CD45/CD105/CD31/CD200 monoclonal or isotype control antibodies (Beckman-Coulter, Villepinte, France), (Becton-Dickinson, Le Pont de Claix, France). Samples were analysed by ADPCyan flow cytometer and Kaluza software (Beckman-Coulter).

### 2.8. Enzyme-Linked Immunosorbent Assay (ELISA)

IL-6 and GDF15 concentrations were evaluated in culture supernatants by using a commercial ELISA kit (R&D Systems, Minneapolis, MN, USA). DKK1 concentration was measured with use of a home-made ELISA kit as previously described [13].

### 2.9. Statistical Analysis

Statistical comparisons involved Student's *t*-test and GraphPad Prism software (La Jolla, CA, USA). Differences were considered statistically significant at *p* < 0.05.

## 3. Results

### 3.1. Transcriptomic Analysis of HD and MM ASCs

**Figure 1:**
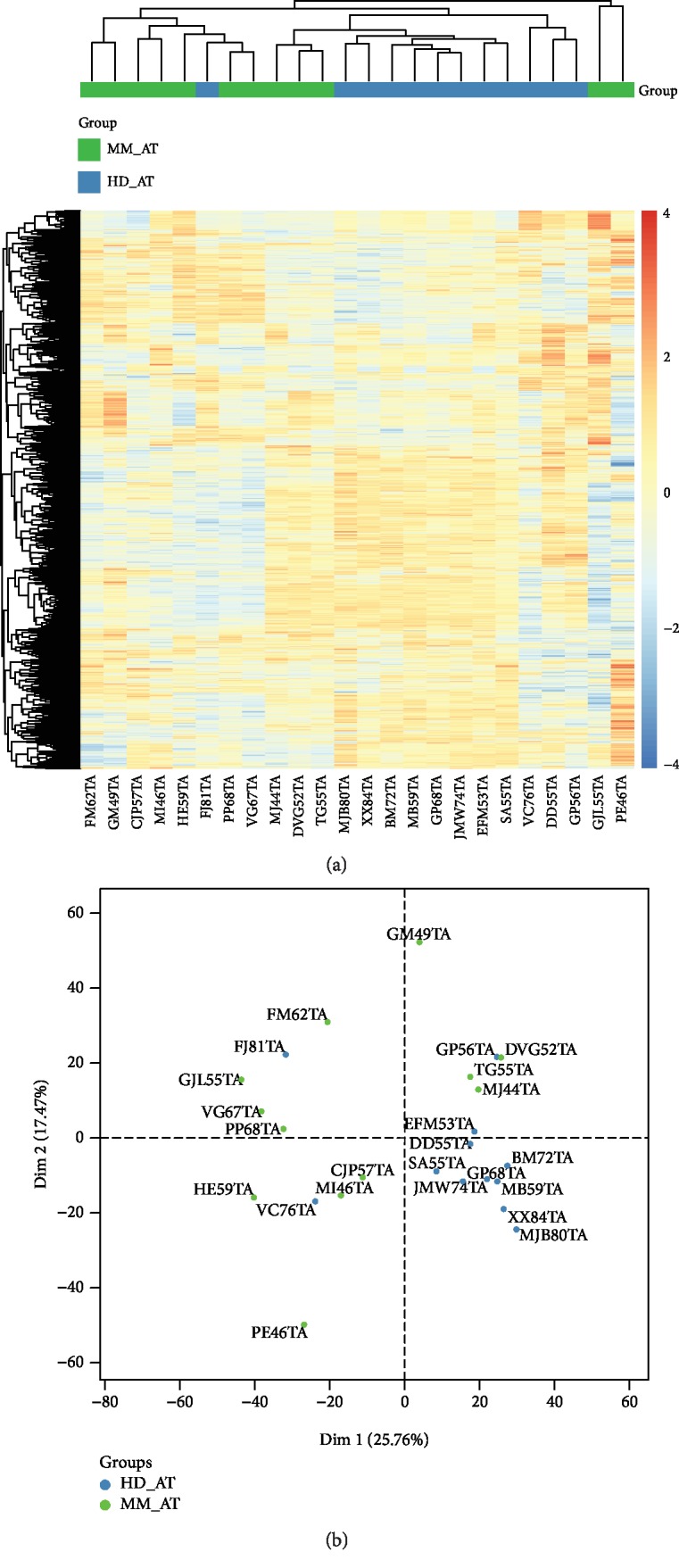
Transcriptomic comparison between multiple myeloma (MM) and healthy donor (HD) adipose stromal cells (ASCs). (a) Heatmap of differentially expressed genes between HD ASCs (blue, *n* = 12) and MM ASCs (green, *n* = 12). (b) Principal component analysis of total gene expression from HD ASCs (blue, *n* = 12) and MM ASCs (green, *n* = 12). Each point corresponds to one patient.

After their expansion, HD/MM ASCs underwent mRNA array analysis. Heatmap classification showed a clustering of HD ASCs except for one donor. MM ASCs were in at least three groups (Figure 1(a)). Nevertheless, we found no sex or age effects, and principal component analysis did not show clear differences (Table 1 and Figure 1(b)). No MM markers were found upregulated in MM ASCs ([Supplementary-material supplementary-material-1]).

### 3.2. Phenotype, Differentiation Potentials, and Hematopoietic Support in HD and MM ASCs as Compared with Autologous MSCs

**Figure 2:**
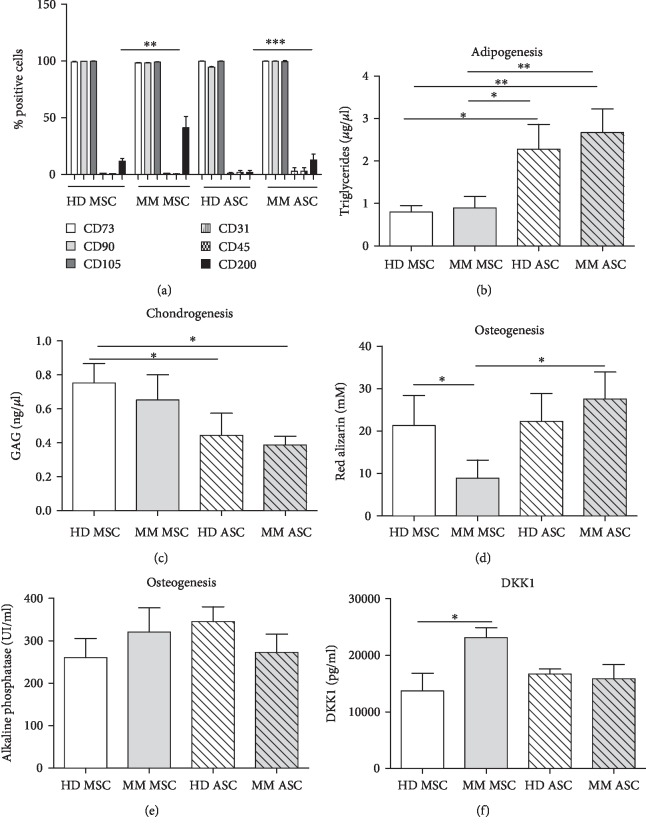
Phenotype and differentiation potential of HD and MM ASCs compared with autologous MSCs. (a) Expression of CD73, CD90, CD105, CD31, CD45, and CD200 in cells after primoculture. Data are mean % positive cells ± SEM (*n* = 12 independent experiments). Adipogenic differentiation with triglyceride dosage (b), chondrogenic differentiation with glycosaminoglycan dosage (c), osteoblastic differentiation with quantification of alizarin red (d), and alkaline phosphatase activity (e) evaluated from all cell sources. Data are the mean ± SEM (*n* = 9 independent experiments). (f) DKK1 secretion from HD/MM ASCs or MSCs. Data are the mean ± SEM (*n* = 8 independent experiments). ^∗^*p* < 0.05, ^∗∗^*p* < 0.01, ^∗∗∗^*p* < 0.001.

**Figure 3:**
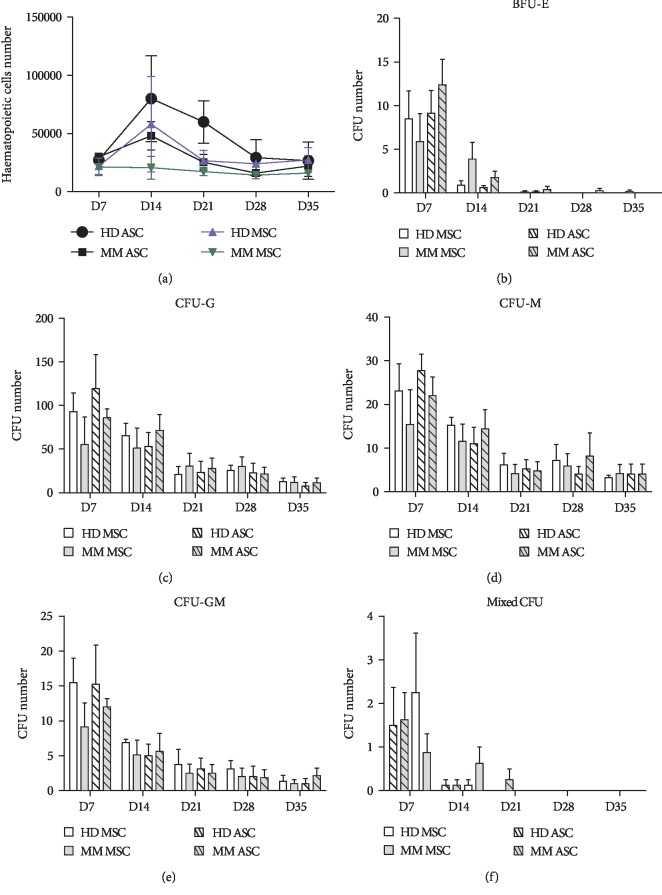
Hematopoiesis support potential of HD and MM ASCs compared with autologous MSCs. CD34+ hematopoietic stem cells were cocultured with HD/MM ASCs or MSCs. At days 7, 14, 21, 28, and 35, nonadherent hematopoietic cells were counted (a) and tested for hematopoietic progenitor content (b) burst forming unit (BFU)-erythroid (BFU-E), (c) colony forming unit (CFU)-granulocytes (CFU-G), (d) CFU-monocytes (CFU-M), (e) CFU-granulocyte macrophages (CFU-GM), and (f) mixed CFU. Data are the mean ± SEM number of CFU (*n* = 4 independent experiments).

As expected, ASCs and MSCs from HDs and MM patients were all positive for CD73, CD90, and CD105 and negative for CD45 and CD31. The only difference among all antigens tested (data not shown) was the significant overexpression of CD200 by MM ASCs and MSCs as compared with their respective normal counterparts (Figure 2(a)). The differentiation ability of MM ASCs and MSC into adipocyte and chondrocyte lineages was similar to that for HD ASCs and MSCs, respectively (Figures 2(b) and 2(c)). Importantly, in contrast to MM MSCs, as already described, osteoblastic differentiation capacity of MM ASCs did not differ from that of their normal counterparts. Moreover, from the same patient, MM ASCs showed a higher mineralization capacity than MM MSCs (Figure 2(d)) in contrast to their alkaline phosphatase activity (Figure 2(e)). In line with mineralization data, secretion of DKK-1 did not differ between MM ASCs and HD ASCs (15.9 × 10^3^ ± 2.5 × 10^3^*vs*. 16.7.10^3^ ± 0.9 × 10^3^ pg/ml, respectively) in contrast to the clear difference between MM and HD MSCs (Figure 2(f)). Functionally, as already described for MSCs, MM and HD ASCs did not differ significantly in their ability to support long-term hematopoietic progenitor cell growth (Figure 3(a)). The total number of hematopoietic colonies generated in methylcellulose after coculture did not significantly differ among all conditions during the study (Figures 3(b)–3(f)).

### 3.3. Comparative Study of Promyeloma Activities from HD and MM ASCs as Compared with Autologous MSCs

**Figure 4:**
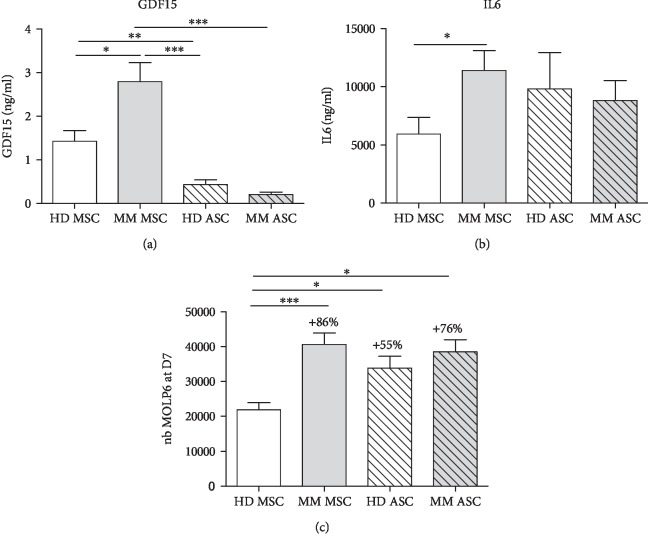
Comparison of promyeloma activities from HD and MM ASCs compared with autologous MSCs. (a) ELISA of GDF15 secretion from HD/MM MSCs and HD/MM ASCs. Data are the mean ± SEM (*n* = 11 independent experiments). (b) ELISA of IL-6 secretion. Data are the mean ± SEM (*n* = 9 independent experiments). (c) Number of MOLP6 MM cells on day 7 after coculture with HD/MM MSCs and HD/MM ASCs. Data are the mean ± SEM (*n* = 9 independent experiments). Represents % increase compared to HD MSC condition. ^∗^*p* < 0.05, ^∗∗^*p* < 0.01, ^∗∗∗^*p* < 0.001.

Among MM markers overexpressed by MM MSCs, MM and HD ASCs did not show excessive secretion of GDF15 as compared with MM MSCs (0.4 ± 0.1 and 0.2 ± 0.05 vs. 2.8 ± 0.4 ng/ml for HD ASCs and MM ASC vs. MM MSCs, respectively, Figure 4(a)). However, ASCs, whether from HD or MM patients, produced as much IL-6 as MM MSCs (9806 ± 3143 and 8806 ± 1710 vs. 11390 ± 1729 ng/ml for HD ASCs and MM ASC vs. MM MSCs, respectively, Figure 4(b)). Furthermore, HD or MM ASCs supported MM MOLP-6 cell line growth as well as MM MSCs as compared with HD MSCs (+55% for HD ASCs, +76% for MM ASCs, and +86% for MM MSCs as compared with HD MSC, Figure 4(c)). This supportive property questions on safety use of ASC at an allogenic (HD ASCs) or autologous (MM ASCs) level.

## 4. Discussion

MM is characterized by bone osteolytic lesions that result from an imbalance between osteoclastic and osteoblastic compartments [4, 26]. Osteoblastic activity is downregulated in MM [16, 27]. MM cells inhibit the differentiation of MSCs into osteoblasts by especially producing the Wnt inhibitor DKK1 [5, 28]. Despite greatly progress in therapeutic strategies, the persistence of bone lesions is still a significant clinical problem and treatment is an unmet medical need. Because MSCs have been successfully used in cellular therapy [29, 30], including bone reconstruction [31, 32], they may have use in MM treatment, in particular during autologous hematopoietic transplant for eligible patients [33]. However, even if MM MSCs are able to correctly support hematopoiesis, we have shown that these cells retain a pathologic signature, including better support of MM cell line growth and a higher expression of IL-6 and GDF15, than do HD MSCs, as well as a differential expression of 145 genes involved in osteogenic differentiation or tumor growth [13, 14].

Stromal cells are found in tissues other than BM, in particular subcutaneous adipose tissue, sharing many characteristics. These cells are a promising source for the regeneration of damaged tissues whatever their origin [34]. ASCs satisfy criteria of MSCs according to the International Society for Cellular Therapy [19, 35]. The ability of ASCs to differentiate into osteoblastic and chondrogenic lineages has been challenged [36], but encouraging results are obtained with bone regeneration therapy [37]. We hypothesized that, in contrast to MSCs, ASCs from MM patients should not be affected by the disease because they are not in direct contact with MM cells. This question remains to be unresolved because the literature data disagree particularly on osteoblastic differentiation [38, 39]. We demonstrated for only mineralization that MM ASCs have a strong ability to differentiate to osteoblastic lineage as compared with MM MSCs, so ASCs from MM patients could be beneficial for MM bone disease, which supports data from Lin and colleagues [39].

Our data confirm previously published results on MM MSC abnormalities [12–14] and demonstrate that ASCs from MM patients and HDs do not show any significant difference in “stromal” immunophenotype, differentiation capacity, and hematopoietic support. These data could suggest that autologous ASCs could be used in MM treatment. However, two elements prevent us from drawing this conclusion. First, we observed a significant overexpression of CD200 by MM ASCs like their MM MSC counterparts [13]. CD200 is a member of the immunoglobulin superfamily and induces an immunoregulatory signal on T cell responses [40, 41]. Its overexpression by MM ASCs may reflect a global immunosuppressive state in MM pathology. Furthermore, MM ASCs supported stroma-dependent MM MOLP-6 cell line growth as well as MM MSCs did. Consequently, the use of MM ASC-based cell therapy in MM could have negative consequences on tumor progression, as suggested in other studies [42]. In addition, HD ASCs have a significantly higher support activity for MM cells as compared with HD MSCs. These important data must alert us to the intrinsic properties of ASCs for allogenic approach-based therapy.

A strength of our study is the use of an “autologous” control from the same participant in order to compare dichotomous HD and MM ASCs with reference to autologous HD or MM MSCs to clearly evaluate the interest of ASCs in MM stem cell therapy.

Taken together, MM ASCs do not exhibit major functional abnormalities, as was observed with MM MSCs. However, MM disease may affect ASCs as suggested by transcriptomic analysis. A functional investigation of genes with altered expression in MM ASCs could give new insights into the pathological role of ASCs highlighted by our results.

## 5. Conclusions

Our data are sufficient to raise suspicions about the safety of the use of ASCs at the autologous or allogenic level for MM treatment. Further studies are required to complete this evaluation before a final decision at the clinical level.

## Figures and Tables

**Table 1:** Characteristics of healthy donor (HD) and multiple myeloma (MM) patients (related to Figure 1).

Patients	Status	Age	Gender	Weight	Bone defects
BM72	HD	44	H	70	∗
DD55	HD	61	H	75	∗
EFM53	HD	63	F	60	∗
FJ81	HD	35	F	70	∗
GP56	HD	60	H	85	∗
GP68	HD	48	H	75	∗
JMW74	HD	42	H	85	∗
MB59	HD	57	F	68	∗
MJB80	HD	36	H	65	∗
SA55	HD	61	H	80	∗
VC76	HD	40	H	90	∗
XX84	HD	32	F	50	∗
PE46	MM	70	H	∗	+
PP68	MM	48	F	∗	−
CJP57	MM	59	H	∗	+
DVG52	MM	64	F	∗	+
FM62	MM	54	H	∗	−
GJL55	MM	61	H	∗	+
GM49	MM	67	F	∗	+
HE59	MM	57	F	∗	+
MI46	MM	70	H	∗	+
MJ44	MM	72	H	∗	−
TG55	MM	61	F	∗	+
VG67	MM	49	H	∗	+

## Data Availability

The data used to support the findings of this study are available from the corresponding author upon request.

## Supplementary Materials

Supplementary Materials Figure S1: list of genes differentially expressed between the 12 MM patients versus HD-derived ASCs.
