# Supplementary material for: Functional Comparison between Healthy and Multiple Myeloma Adipose Stromal Cells
Source: Stem Cells Int. 2020 Mar 5;2020:4173578. doi: 10.1155/2020/4173578 (PMC7077052; doi:10.1155/2020/4173578)
Supplement: Supplementary Materials — Figure S1: list of genes differentially expressed between the 12 MM patients versus HD-derived ASCs. [file 4173578.f1.pdf]

Fig.S1

hgnc_symbol	FC	adj,P,Val
ZIC1	29,9468358	3,25E-12
ZIC1	8,92976686	1,24E-10
SCARA5	7,11830135	0,020981361
CHI3L1	6,94684579	0,021848595
STEAP4	6,33465325	0,005135315
IGDCC4	5,36836205	3,60E-05
LINC01139	5,12654792	7,63E-07
COL21A1	4,93443113	0,003778994
SCARA5	4,92217859	0,031808641
CHI3L1	4,91784554	0,04068759
CCRL1P1	4,91513289	0,001456342
ACKR4	4,91513289	0,001456342
PLA2G2A	4,75508688	0,02557513
RIPOR3	4,6855866	0,007002547
H19	4,57852555	0,035905081
TNFSF10	4,53739769	0,0359134
CPXM1	4,49901016	0,018033398
TNFSF10	4,36386792	0,014822794
COLEC12	4,24269347	0,02225264
SLC39A8	4,22289354	0,009500521
OGN	4,21387642	0,020801429
EXOSC7	4,13166809	0,030382113
CLEC3B	4,13166809	0,030382113
CXCL6	4,12577862	0,029279699
TNFSF10	4,06467643	0,008737424
SHOX2	3,93081951	0,002569507
SIM1	3,87370239	0,002265585
C1S	3,87267942	0,035905081
CLU	3,76726118	0,03794651
CDON	3,7609255	0,005012894
PLAU	3,70709775	0,006311287
FMO1	3,66586676	0,012738187
SLC39A8	3,59875535	0,010921568
MEOX2	3,49460728	0,006276374
ZIC1	3,46516773	2,94E-05
LSAMP	3,43569361	0,005135315
ADAMTS5	3,35239437	0,008638262
PDGFD	3,34582668	0,003778994
OLFML2A	3,3274322	0,003167173
TSHZ2	3,31898226	0,003778994
LRRN4CL	3,31855994	0,037066434
OGN	3,30133978	0,020065962
TGFBR3	3,26606175	0,003167173
PTGFR	3,16884863	0,013101053
PDE1A	3,15603823	0,003778994
FGL2	3,1270578	0,028698184
PDE1A	3,11255562	0,00147887
PLAU	3,09331401	0,020666244
FBLN1	3,08019008	0,039145159

AKAP12	3,07938853	0,009227265
PCOLCE2	3,03848049	0,001191464
CXCL1	3,02194545	0,037580921
PDE1A	2,98737778	0,003167173
APOD	2,93341115	0,044354921
PDGFRL	2,89694047	0,023081694
LINC02511	2,87912423	0,030134614
LRIG3	2,87880305	0,005361796
CYP7B1	2,86826076	0,010167695
TMEM30B	2,8672913	0,000567164
PDE1A	2,85720646	0,005361796
CHRDL1	2,84047878	0,049550944
SIM1	2,84020958	0,001191464
MAF	2,83298878	0,02557513
TGFBR3	2,82437111	0,007002547
STEAP4	2,81342211	0,007605967
GIPC2	2,80066219	0,003167173
TSHZ2	2,78706681	0,008012402
GPR39	2,76243631	0,008389791
SRPX	2,76147595	0,013437555
OMD	2,75616592	0,017806793
SNED1	2,74573847	0,044846817
SHOX2	2,73276018	0,006499649
SOD2	2,71612177	0,019583992
PDE1A	2,71578524	0,006276374
DCLK1	2,7000751	0,029279699
DCLK1	2,69412582	0,024354186
PTPRD-AS1	2,692947	0,020682839
KCNJ15	2,68645224	0,016043971
XPNPEP2	2,68189186	0,039753468
ZEB1	2,67167599	0,004686967
RASD1	2,65038986	0,036460868
RIPOR2	2,64684554	0,005713996
DCLK1	2,64643802	0,026654113
ETV1	2,64367797	0,049096849
CXCL12	2,64180156	0,010921568
PTPRD	2,63939735	0,030134614
S100A4	2,63923403	0,010665507
CREB5	2,63794464	0,002265585
PTGER3	2,6346815	0,015395119
PTPRD	2,61222751	0,037772848
HOTAIR	2,60464407	0,002265585
ADAMTS5	2,58881096	0,015938008
FGL2	2,57941057	0,044250747
SCARA3	2,56505335	0,009009044
IL17RD	2,56319115	0,030382113
PDGFD	2,53034046	0,008166513
SOD2	2,52076144	0,030382113
NT5E	2,51956559	0,003573357
TRH	2,51210289	0,04149936

CXCL12	2,49744978	0,013457542
ADAMTS5	2,49720436	0,015909903
STEAP4	2,47578278	0,020330717
ADAMTS15	2,47297299	0,036460868
BTN3A3	2,47185758	0,011547111
CREB5	2,46669232	0,003778994
TJP2	2,44975823	0,008442413
202085_at	2,44975823	0,008442413
IL6ST	2,44279272	0,026170493
LSP1	2,44253009	0,018877608
TSHZ2	2,4287565	0,037580921
AKAP12	2,40880848	0,015909903
NCALD	2,39695004	0,005361796
KLF3	2,37892173	0,010665507
ZNF521	2,37759216	0,04478322
EPB41L3	2,36978285	0,04478322
IL1R1	2,36223913	0,012960391
TMEM35A	2,36057061	0,012960391
TRERF1	2,35932916	0,008638262
PDE7B	2,3485274	0,006647412
DENND2A	2,33322298	0,006647412
CCL8	2,3267133	0,023098928
FAM20A	2,31404049	0,024270135
MAFB	2,31001803	0,045005851
SULF2	2,2875399	0,031426752
SCARA3	2,27496611	0,015395119
DNM1	2,26339916	0,046931852
FGF18	2,26239051	0,04478322
TSHZ2	2,2605117	0,024270135
GREM2	2,25916986	0,047979011
SOCS3	2,23867341	0,026214035
DCLK1	2,23544077	0,04149936
ARHGEF3	2,22657071	0,024354186
KCND2	2,22544138	0,024221152
LGR4	2,2221427	0,006647412
ZNF436	2,20924198	0,015395119
FAM20A	2,20588627	0,037580921
SULF2	2,1997055	0,044354921
GUCY1A2	2,19812413	0,044354921
MCC	2,19286783	0,015395119
TNXA	2,18779922	0,044354921
TNXB	2,18779922	0,044354921
GFRA1	2,18143382	0,010004198
PMAIP1	2,17769313	0,030382113
AKAP12	2,17729182	0,026380453
ZFP36L2	2,17501764	0,031426752
PRRX1	2,1718123	0,021848595
215223_s_at	2,16867338	0,047402599
SOD2	2,16867338	0,047402599
SLC2A5	2,16242772	0,016785421

TCEAL7	2,15790641	0,03828766
FAM3C	2,1539948	0,006702
CPZ	2,15035423	0,047807622
GPR78	2,15035423	0,047807622
GASK1B	2,14279972	0,017510044
AXIN2	2,14234333	0,009009044
AHNAK2	2,13932703	0,0238667
CAMK2N1	2,13701388	0,040507605
ZFHX4-AS1	2,13642818	0,013101053
ELFN1	2,13446961	0,012960391
EBF2	2,13004332	0,03828766
PTGFR	2,12720921	0,018855648
ZNF385D	2,12495506	0,025760319
DENND2A	2,11410053	0,013976681
C1RL	2,11348601	0,035320026
BTN3A2	2,11345066	0,011856422
RASSF2	2,10126655	0,015909903
ADAMTS5	2,09876171	0,046157453
C17orf58	2,07834809	0,029094357
SLC9A9	2,07502055	0,013498642
C10orf90	2,07402643	0,02225264
CD44	2,06617832	0,017795471
OMD	2,06441217	0,04679665
COL8A2	2,05551112	0,013101053
IL6ST	2,05153127	0,037580921
CHURC1	2,05074248	0,045827101
ELOVL2	2,04452527	0,02058644
GIPC2	2,04139666	0,030382113
SAMHD1	2,03848245	0,03037556
PMAIP1	2,03235496	0,022637296
CARD10	2,03090126	0,035248773
MYO1D	2,02969717	0,026170493
KCNK2	2,02676894	0,012960391
PTGER3	2,02660094	0,037580921
SOCS3	2,01017884	0,045005851
KCTD12	2,00983296	0,047979011
EML1	2,00185849	0,013498642
ZNF436	2,00059984	0,04348027
ANKRD33B	2,000044	0,030367087
ARHGAP6	1,99860169	0,038620226
HRH1	1,98991737	0,026380453
ANK2	1,98584407	0,047979011
LSAMP	1,98580279	0,033326704
IL6ST	1,98421127	0,026380453
SELENBP1	1,97398882	0,047599154
MAN1A1	1,96975609	0,01779173
209355_s_at	1,96007794	0,03828766
PLPP3	1,96007794	0,03828766
SFR1	1,95641279	0,044250747
SLC44A1	1,95177347	0,015395119

VSTM4	1,94692793	0,012960391
CTSLP8	1,94376034	0,020330717
RUBCNL	1,94144834	0,029684154
STMN3	1,93473206	0,044250747
ERG	1,93279202	0,046157453
EBF1	1,92844131	0,021111024
GAS1	1,92649274	0,018855648
GCLM	1,91482895	0,03909725
BTN3A3	1,91346455	0,03828766
TSHZ2	1,90796903	0,029501573
RRAD	1,90638153	0,044354921
PROCR	1,90343219	0,026380453
ACE	1,90019294	0,049997428
SECTM1	1,89988517	0,04149936
ADCY3	1,89603259	0,045005851
KLF4	1,89163252	0,04149936
APOL3	1,8835642	0,039050336
MAN1A1	1,88253803	0,046149416
CA12	1,88020179	0,040316404
ZFP36L2	1,87910478	0,018253442
PDE1A	1,8774423	0,030382113
PRRX1	1,87297874	0,033470488
EML1	1,87290716	0,023081694
PROS1	1,87235679	0,03828766
GALNT16	1,86087921	0,033808209
IL1R1	1,84994768	0,049649966
RSRC1	1,84965909	0,032115746
DIRC3	1,8494934	0,046653228
GLI3	1,84847509	0,030382113
CD44	1,84640355	0,037231257
CDC14B	1,83781918	0,022637296
JAK1	1,8239494	0,04149936
SMURF2	1,8160758	0,04348027
CD44	1,81535073	0,035248773
CD47	1,80443532	0,029410538
IL6ST	1,79909937	0,028698184
RNASE4	1,78062866	0,046366154
205158_at	1,78062866	0,046366154
ANG	1,78062866	0,046366154
IRAK3	1,77631641	0,04348027
SMKR1	1,77539049	0,040111659
REV3L	1,76964273	0,04149936
CD44	1,76648816	0,030367087
PTPRG	1,75989493	0,033719309
PRRX1	1,75775559	0,04348027
RAC1	1,75103171	0,046653228
SLC44A1	1,7260569	0,036460868
ZCCHC24	1,72276033	0,030382113
ARHGAP24	1,71515717	0,030382113
KCNJ18	1,69694346	0,045005851

KCNJ12	1,69694346	0,045005851
